# Expression of *OsMYB55* in maize activates stress-responsive genes and enhances heat and drought tolerance

**DOI:** 10.1186/s12864-016-2659-5

**Published:** 2016-04-29

**Authors:** José A. Casaretto, Ashraf El-kereamy, Bin Zeng, Suzy M. Stiegelmeyer, Xi Chen, Yong-Mei Bi, Steven J. Rothstein

**Affiliations:** Department of Molecular and Cellular Biology, University of Guelph, Guelph, ON N1G 2W1 Canada; Syngenta Biotechnology Inc., Research Triangle Park, NC 27709 USA; University of California, Agriculture and Natural Resources, Cooperative Extension - Kern County, Bakersfield, CA 93307 USA; Expression Analysis, Inc., Durham, NC 27713 USA

**Keywords:** Drought, Heat, Maize, MYB, Stress, Zea mays

## Abstract

**Background:**

Plant response mechanisms to heat and drought stresses have been considered in strategies for generating stress tolerant genotypes, but with limited success. Here, we analyzed the transcriptome and improved tolerance to heat stress and drought of maize plants over-expressing the *OsMYB55* gene.

**Results:**

Over-expression of *OsMYB55* in maize decreased the negative effects of high temperature and drought resulting in improved plant growth and performance under these conditions. This was evidenced by the higher plant biomass and reduced leaf damage exhibited by the transgenic lines compared to wild type when plants were subjected to individual or combined stresses and during or after recovery from stress. A global transcriptomic analysis using RNA sequencing revealed that several genes induced by heat stress in wild type plants are constitutively up-regulated in OsMYB55 transgenic maize. In addition, a significant number of genes up-regulated in OsMYB55 transgenic maize under control or heat treatments have been associated with responses to abiotic stresses including high temperature, dehydration and oxidative stress. The latter is a common and major consequence of imposed heat and drought conditions, suggesting that this altered gene expression may be associated with the improved stress tolerance in these transgenic lines. Functional annotation and enrichment analysis of the transcriptome also pinpoint the relevance of specific biological processes for stress responses.

**Conclusions:**

Our results show that expression of *OsMYB55* can improve tolerance to heat stress and drought in maize plants. Enhanced expression of stress-associated genes may be involved in OsMYB55-mediated stress tolerance. Possible implications for the improved tolerance to heat stress and drought of OsMYB55 transgenic maize are discussed.

**Electronic supplementary material:**

The online version of this article (doi:10.1186/s12864-016-2659-5) contains supplementary material, which is available to authorized users.

## Background

It is anticipated that the demand for cereals will increase by 70 % by 2050 given the increased food production needed to sustain a growing population, with the largest increases required in many low-income countries [1]. This pressure for more food is complicated by the negative effects of global climate change on the efficient use of water, energy and land for major crops [2]. Extreme temperatures and water deficits are among the most serious climatic factors limiting crop production. On average, our planet’s surface temperature has registered an increase of 0.6 °C over the past century and a further increase of 2 to 4 degrees has been predicted by the end of the present century [3]. Although this may be beneficial for a few crops in certain high latitude regions, overall productivity will be negatively affected because of higher temperatures during the growing season and more severe and longer droughts [4]. Thus, under this future scenario, there is a need to expand breeding programs and biotechnology strategies to improve growth performance of major crops by introducing varieties with higher yield and less vulnerability to diseases and other environmental stresses.

High temperatures adversely affect germination and water relations, shorten developmental stages and cause perturbation of photosynthesis-related processes such as light perception, carbon fixation and respiration, leading to heat-induced yield loss in cereals [5]. Even with sufficient moisture, higher temperatures both at day and night have an effect on yield potential. For maize for instance, temperatures above 35 °C (above the optimum daytime range of 25–30 °C) will affect vegetative and reproductive growth, from germination to grain filling [6]. At the cellular level, the major consequences of high temperatures are: (1) alteration of membrane fluidity affecting membrane function which has an impact on photosynthesis and respiration and (2) onset of oxidative damage caused by the heat-induced imbalance of photosynthesis and respiration, production of reactive oxygen species (ROS) and reduced antioxidant activity [7]. Similar to reacting to drought and salinity, plants deploy various protection mechanisms to endure heat stress (HS) including scavenging of ROS, production of antioxidants, accumulation of compatible solutes and activating signaling cascades leading to the synthesis of molecular chaperones such as heat-shock proteins (HSPs) and late embryogenesis abundant (LEA) proteins. These chaperones would prevent denaturation of existing proteins, misfolding of newly synthesized proteins and maintaining membrane stability [8]. All these response mechanisms have been considered in strategies for improving heat tolerance through modern breeding protocols and biotechnological approaches, though up to now their success has been limited.

Studies on heat response and heat tolerance have largely focused on heat shock proteins (HSPs) and transcriptional activation of their genes by heat shock transcription factors (HSFs) [8]. Positive correlations between expression of HSPs and HS tolerance have been reported [9–12]. Nonetheless, besides HSPs, other pathways can be potentially manipulated to improve thermotolerance. It has been suggested that part of the response to HS is mediated by the action of plant growth regulators such as cytokinin [13], methyl jasmonate [14], salicylate [15] and brassinolides [16]. Similarly, other molecules such as proline, glycine-betaine, polyamines or signal molecules (e.g. ABA, calcium, hydrogen sulfide) can aid in maintaining cell membrane integrity, ion homeostasis and increased photosynthesis, either when applied exogenously [17] or by manipulating their biosynthesis [18–20]. Although differences exist between responses to HS and drought when imposed individually [21], the above examples indicate that reactions to HS often involve interconnected networks and defense mechanisms shared with other stress responses. This is further evidenced by the protective role of HSFs and HSPs against more than one abiotic stress condition [22–24].

Tolerance to heat is generally characterized by transcriptional activity leading to synthesis of protective components as mentioned above. A transcriptome analysis performed in wheat, revealed the induction of a large number of transcription factors following heat treatment, including members of the HSF, AP2, bHLH, bZIP, MYB, NAC and WRKY and Zn-finger gene families [25]. Interestingly, members of the same gene families were recently identified in a transcriptome study in rice [26]. Data of the latter study showed 31 and 21 out of the 46 differentially expressed MYB genes to be up-regulated one and eight hours after heat stress, respectively, suggesting a central role of these genes in the transcriptional response to HS. Plant MYB transcription factors, most of the R2R3 type, regulate numerous processes during the plant life cycle including responses to environmental stresses [27]. Despite the large number of MYB genes in plant species, only few have been proposed to have a role in heat tolerance. In *Arabidopsis*, mutants for the *MYB68* gene show reduced growth under high temperature compared to WT [28]. Recently, it has been reported that constitutive expression of the tomato *LeAN2* gene encoding a MYB transcription factor causes anthocyanin accumulation and confers enhanced tolerance to HS by maintaining a functional photosynthetic apparatus and a higher non-enzymatic antioxidant activity [29]. In a previous study, our group described that over-expression of *OsMYB55* in rice stimulated amino acid metabolic pathways crucial for normal plant growth and development, resulting in improved plant tolerance to HS during vegetative growth and decreasing the negative effect of high temperature on grain yield [30].

In this work, we analyzed the effect of over-expressing *OsMYB55* in maize, an important crop with different photosynthetic mechanism than rice (C4 vs. C3) but adapted to a similar climate. We performed a wide transcriptome analysis to investigate the genes and pathways affected by HS and *OsMYB55* over-expression. In addition, because a combination of HS and drought better resembles a predicted scenario of climate change, we assessed the individual and additive effects of these stress conditions on the growth of WT and transgenic plants.

## Results

### Phenotypic responses to heat stress

**Fig. 1:**
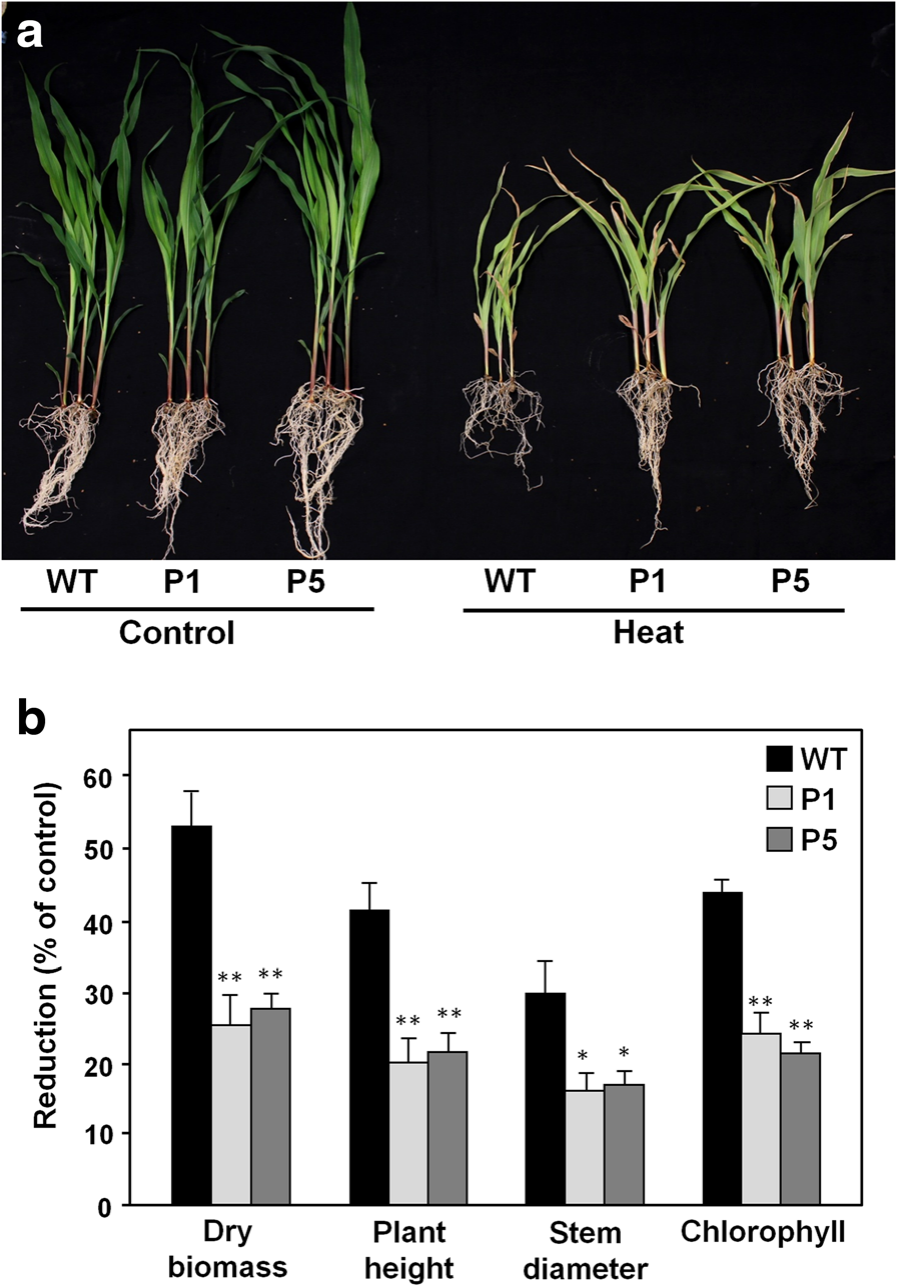
Tolerance to high temperature of maize seedlings over-expressing *OsMYB55.* **a** Wild type (WT) and over-expression transgenic (P1, P5) plants were grown at control temperature (29 °C / 23 °C day/night) until the second leaf stage. Plants were then moved to high temperature regime (42 °C /35 °C) for five days. Control plants were maintained at normal temperature. **b** Measurement of total dry biomass, plant height, stem diameter and chlorophyll content after five days of heat treatment, expressed as reduction compared to control plants. Bars represent means ± SE. Asterisks indicate significant differences between transgenic (P1, P5) and wild type (WT) plants (*t*-test, * *P* < 0.05, ** *P* < 0.01)

Maize plants over-expressing the full-length cDNA of the *OsMYB55* gene and representing ten single-copy transgenic lines were generated. Four transgenic lines were randomly chosen to carry out the experiments described in this study and the expression levels of the transgene were determined (Additional file 1). Seedlings from WT and two OsMYB55 transgenic lines were used to make an initial assessment of their growth in response to high temperature. Plants entering the three leaf-stage were moved from 29 to 42 °C (daylight temperature) and response to HS was evaluated by determining total dry biomass, plant height, stem diameter and chlorophyll content. Growth reduction of transgenic plants was less affected than WT after five days of heat treatment, reflected by a small reduction of dry biomass, plant height, and stem diameter (Fig. 1). In addition, smaller decrease in chlorophyll content in OsMYB55 lines indicated reduced initial leaf damage to the leaves compared to WT (Fig. 1).

### Transcriptome analysis of transgenic maize under heat

Because of the significant growth differences observed in response to high temperature, a similar experiment was devised to compare the transcriptomes of one transgenic line (P4, which presented median levels of transgene expression among the selected lines; Additional file 1) and WT plants. Transcript profiling (RNA-Seq) was performed using mRNA from leaves, stems and roots of plants grown under normal and high temperature conditions. Over 80 % of the filtered reads aligning to the reference genome (Additional file 2). Of 36,603 genes, 23,778 genes remained after filtering. Comparisons were made within leaf, stem and root samples of each genotype between control and HS conditions and between genotypes. These datasets are listed in Additional files 3, 4 and 5. The number of differentially expressed genes (DEG; fold-change greater than 2, *P* < 0.05) is summarized in Additional file 6. With these criteria, in all three tissues, more than half of the DEG between control and HS were common for WT and the OsMYB55 line. In leaves, 885 and 946 were significantly differentially expressed only in WT and only in the transgenic line, respectively, and 2,070 were common DEG for both genotypes (Additional file 6a). Lower number of up- and down-regulated entities was found for stems (Additional file 6b) and roots (Additional file 6c). Comparisons between the genotypes showed reduced number of DEG for plants under control or HS (Additional file 7).

**Fig. 2:**
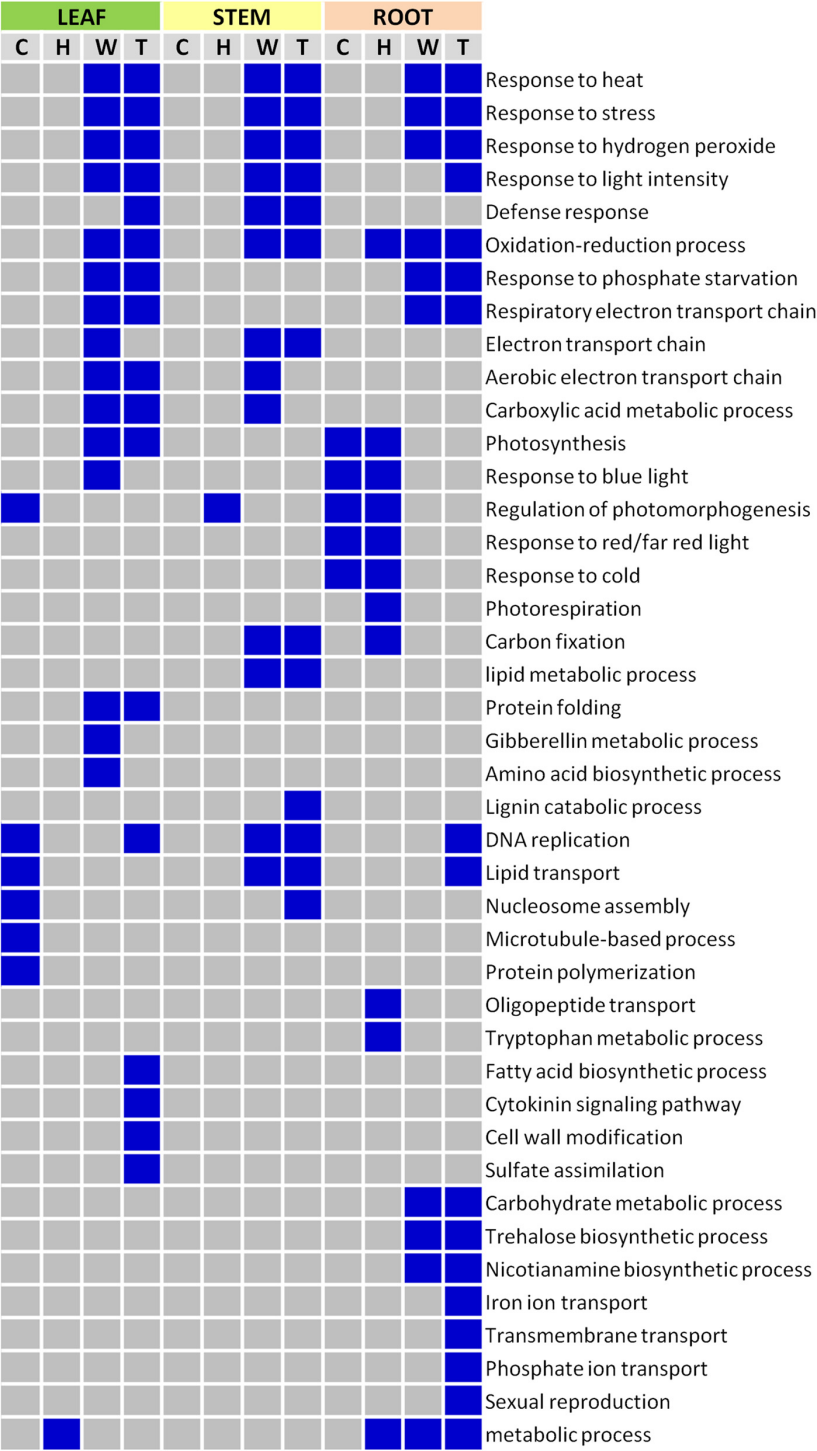
Enrichment of Biological Process GO Terms for DEG. Enriched terms by tissue and category (C, control; H, heat stress; W, wild type; T, transgenic line). Blue indicates statistically significance, FDR < 0.05

Gene Ontology (GO) functional enrichment analysis was performed with the gene lists for each tissue from the comparisons between conditions (control and heat) and between genotypes (WT and OsMYB55 line). GO terms for biological processes are shown in Fig. 2 and are plotted as a heat map to easily visualize common functionality across experimental conditions. In leaf samples, six GO terms were enriched in the control condition when comparing OsMYB55 line with WT, whereas only one GO term (metabolic process) was enriched in the heat treatment comparison. When analyzing the genes up-regulated in leaves in response to heat, 15 GO terms were enriched in WT whereas 17 GO terms were enriched in the transgenic line. Of those, 11 were common in both genotypes (response to heat, response to stress, response to hydrogen peroxide, response to light intensity, oxidation reduction process, phosphate starvation, respiratory electron transport chain, aerobic electron transport chain, carboxylic acid metabolic process, photosynthesis and protein folding). Most of these terms were also enriched in the same comparisons for the stem and root samples, in either genotype or both (Fig. 2). Other enriched terms in stem samples were associated with carbon fixation, lipid metabolic process, DNA replication and lipid transport in both genotypes, plus those related to lignin catabolic process and to nucleosome assembly in the transgenic line. GO terms specifically enriched in roots included carbohydrate metabolic process, trehalose biosynthetic process, nicotianamine biosynthetic process, ion transport, phosphate transport, transmembrane transport, sexual reproduction, with the last four only enriched in the transgenic line (Fig. 2).

To have a general idea of the pathways affected by differences in gene expression, we used GOseq and the same methodology for GO Term enrichment to identify enriched pathways. In leaves, two pathways related to synthesis of gibberellins were enriched in both conditions, and pathways associated with biosynthesis of conjugated cytokinin and branched amino acids were also enriched under HS. In addition, heat treatment caused enrichment of sulfate assimilatory pathways and phosphate acquisition in the transgenic line (Additional file 8). In stems, a triaglycerol degradation pathway was enriched in the transgenic line. In roots, the transgenic line presented significant representation of pathways associated to photosynthesis, carbon reduction and gluconeogenesis in either treatment condition and five enriched biosynthetic pathways for flavonoids and anthocyanidins when the OsMYB55 line was compared to WT in the HS condition (Additional file 8).

### Several heat-induced genes in WT plants are constitutively up-regulated in OsMYB55 transgenic maize

The response to heat treatment by the two genotypes was further analyzed by focusing in the transcriptome obtained from leaf samples. Evaluation of the DEG lists for treatment and genotype comparisons revealed that most of the significantly up-regulated genes in the transgenic line under control conditions are also highly up-regulated in WT plants in response to HS, though only about half of them presented significant P-value (Additional file 9). In addition, six of those genes were significantly up-regulated in the transgenic line by HS. Annotation for most of these entities obtained from the MaizeGDB shows that several of them are associated to stress responses or regulation of transcription (e.g. transcription factors). Comparison of DEG in the OsMYB55 line with those in WT under control conditions showed that 47 entities displayed significant differential expression in at least two of the organs sampled (considering a fold-change ≥ 2 in at least one sample; Additional file 10).

**Fig. 3:**
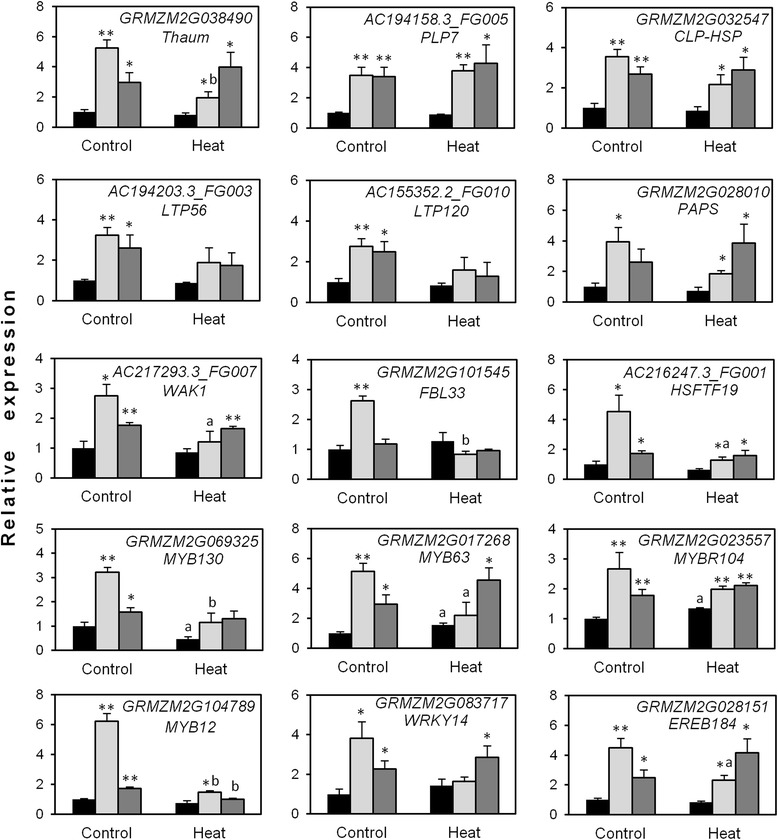
Expression of selected genes identified by RNAseq in transgenic lines. Relative transcript levels (qRT-PCR) of GRMZM2G038490 (Thaumatin); AC194158.3_FG005 (Patatin-like phospholipase); GRMZM2G032547 (Heat shock protein); AC194203.3_FG003 (LTPL56); AC155352.2_FG010 (LTPL120); GRMZM2G028010 (adenosine phosphosulfate reductase); AC217293.3_FG007 (wall associated kinase receptor-like); GRMZM2G101545 (F-box and LRR protein 33); AC216247.3_FG001 (HSFTF19); GRMZM2G069325 (MYB55); GRMZM2G017268 (MYB63); GRMZM2G023557 (MYB-related-transcription factor 104); GRMZM2G104789 (MYB12); GRMZM2G083717 (WRKY80) and GRMZM2G028151 (EREB184). Bars indicate mean relative expression values ± SE Asterisks indicate significant differences between transgenic (P4, P5; grey bars) and wild type (WT, black bars) plants and letters between heat-treated and control plants (*t*-test, * and *a P* < 0.05, ** and *b P* < 0.01)

A group of genes from tables in Additional files 9 and 10 were selected to validate their expression in two independent OsMYB55 lines. They encode a thaumatin, a patatin-like phospholipase, a heat shock protein, two lipid transfer protein family members, a phosphoadenosine phosphosulfate reductase, a wall associated kinase receptor-like protein, an F-box and leucine-rich repeat protein, a heat shock transcription factor, four MYB transcription factors, a WRKY transcription factor and an AP2-like ethylene responsive transcription factor. Of those 15 selected genes, 13 showed significant up-regulation in both transgenic lines compared to WT in the control condition, whereas only 7 in the heat treatment condition (Fig. 3). Two MYB transcription factors (MYB63 and MYBR104) were induced in the WT in response to heat. Also, in concurrence with the RNAseq data (Additional file 3), some genes up-regulated in one transgenic line (compared to WT) in the heat stress comparison displayed decreased expression when comparing heat and control samples (of the same transgenic line). Despite this, most genes showed higher expression in both transgenic lines compared to WT under HS (Fig. 3). These results confirm the transcript abundance of DEG from the RNAseq assay and suggest that the profile of induced genes in the OsMYB55 lines may be related to the improved heat tolerance.

### Several genes up-regulated in OsMYB55 transgenic maize are also associated with drought responses

The possible role of OsMYB55 in modulating expression of genes involved in responses to abiotic stress especially drought was further investigated by analyzing GO terminology of the DEG comparison under control conditions. Out of the 173 genes up-regulated in the transgenic maize line (Additional file 7), 18 genes were classified by AgriGO as belonging to GO:0009628 (response to abiotic stimulus; Additional file 11). In addition, 24 genes were identified as being up-regulated at least in one drought transcriptome study in maize reported in the literature (Additional file 11). Moreover, a literature search provided further information for 21 genes in maize or for their orthologs in rice or other species. In those cases, a significant number of genes have been described to be induced by drought or to confer stress tolerance when over-expressed (Additional file 11). In that gene list, nine of them encode transcription factors and 11 encode chromatin-related proteins (e.g. histones and minichromosome maintenance complex subunits). Other genes are associated with hormone signaling, oxidative stress and defense responses.

### OsMYB55 transgenic maize shows tolerance to heat and drought stress

**Fig. 4:**
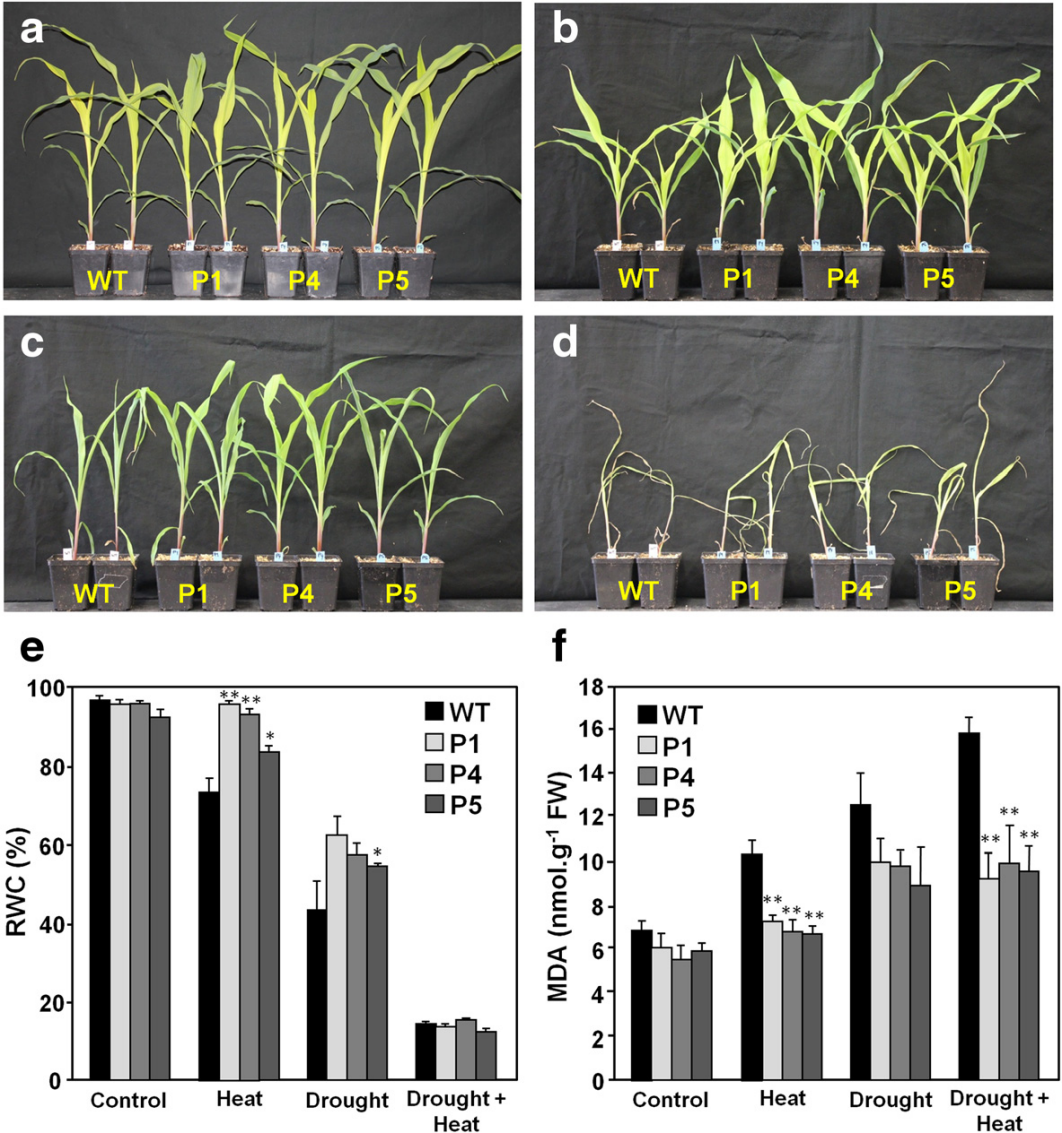
Phenotype of maize OsMYB55 transgenic plants subjected to drought and heat stress. Five-week old wild type (WT) and over-expression plants (P1, P4, P5) were grown under **a** control conditions, **b** water withholding, **c** high temperature (42 °C / 35 °C) or **d** subjected to water withholding and high temperature as described in methods. Picture was taken five days after treatments. Control plants were watered and maintained at normal temperatures (29 °C / 23 °C) during the experiment. **e** Relative water content at the end of the treatment. **f** Malondihaldehyde (MDA) accumulation in leaves as indication of stress-induced oxidative damage. Bars represent mean values ± SE (*n* = 6). *P*-values (Student’s *t*-test) are indicated: **P < 0.005 and *P < 0.05

The above results of the transcriptome analysis led us to further evaluate the response of plants expressing *OsMYB55* to HS, drought and the combination of these stresses. Five-week-old plants of three independent lines and WT plants grown under controlled conditions were subjected to water withholding or high temperature treatment or both for five days before evaluation of growth and physiological parameters. Even though visual differences were noticeable between the genotypes (Fig. 4a-d), variations in some parameters were statistically significant only in certain cases. All three treatments caused reduction of water content in WT plants however less significant reduction was observed in transgenic lines under HS (Fig. 4e). Plant growth and chlorophyll content were also reduced in all genotypes in all three conditions (Table 1). Significant differences in plant height and leaf damage were observed between the transgenic lines and WT under HS, whereas significant differences in stem diameter, chlorophyll content and leaf damage were observed between all three genotypes under combined stress. Only one transgenic line (P1) displayed more fresh and dry biomass than WT under HS. In the drought treatment, all lines presented higher chlorophyll content and dry biomass than WT (Table 1). Consistent with the observed leaf damage, all three transgenic lines accumulated less malondihaldehyde (MDA) than WT in all stress conditions, although differences were only significant under HS and combined stress (Fig. 4f). After the treatments, the remaining plants from the drought and HS groups were allowed and were able to recover (transferred to control temperature and re-watered for seven days). While all genotypes presented similar growth in the control group, the three transgenic lines exhibited higher biomass than WT in both the drought and HS groups (Additional file 12), magnifying the effects of the stress treatments and the differences between the genotypes. Plants in the combined HS and drought treatment group were not considered in this case because the additive effect of the same intensity and duration of stresses was too severe to allow plants to fully recover.

**Table 1:** Growth and damage parameters measured in plants subjected to heat and combined heat and drought stress

Treatment	Genotype	Height* (cm)	Stem diameter* (mm)	Damaged leaves*	Chlorophyll Content* (index units)	Fresh biomass* (g)	Dry biomass* (g)
Control	WT	59.1 ± 1.07^a^	8.2 ± 0.16^a^	0.0 ± 0.00^a^	19.3 ± 0.45^a^	9.0 ± 0.53^a^	0.74 ± 0.05^a^
	P1	59.2 ± 1.43^a^	8.1 ± 0.17^a^	0.0 ± 0.00^a^	19.9 ± 0.70^a^	9.2 ± 0.76^a^	0.75 ± 0.06^a^
	P4	58.8 ± 1.12^a^	8.4 ± 0.50^ab^	0.0 ± 0.00^a^	21.7 ± 1.12^a^	11.0 ± 0.39^a^	0.85 ± 0.06^a^
	P5	60.2 ± 1.10^a^	8.9 ± 0.38^b^	0.0 ± 0.00^a^	20.6 ± 1.36^a^	10.9 ± 0.36^a^	0.80 ± 0.05^a^
Heat	WT	44.8 ± 1.61^b^	5.9 ± 0.31^c^	2.1 ± 0.35^b^	7.7 ± 0.81^be^	5.3 ± 0.47^b^	0.41 ± 0.05^b^
	P1	49.6 ± 1.60^c^	6.5 ± 0.25^cd^	0.8 ± 0.32^c^	10.0 ± 1.15^bc^	7.4 ± 0.56^c^	0.61 ± 0.06^c^
	P4	48.9 ± 1.14^c^	6.7 ± 0.25^d^	0.4 ± 0.18^c^	8.3 ± 0.99^b^	5.7 ± 0.52^b^	0.45 ± 0.05^b^
	P5	47.2 ± 0.79^bc^	6.9 ± 0.36^d^	0.9 ± 0.20^c^	9.0 ± 1.32^b^	5.7 ± 0.73^b^	0.44 ± 0.05^b^
Drought	WT	49.0 ± 1.17^c^	5.8 ± 0.15^c^	0.0 ± 0.00^a^	12.7 ± 0.67^c^	4.2 ± 0.34^d^	0.31 ± 0.02^d^
	P1	53.0 ± 1.01^c^	6.5 ± 0.15^d^	0.0 ± 0.00^a^	18.1 ± 0.68^a^	5.0 ± 0.30^b^	0.44 ± 0.03^b^
	P4	51.3 ± 0.60^c^	6.6 ± 0.37^d^	0.0 ± 0.00^a^	16.5 ± 1.12^a^	4.4 ± 0.39^bd^	0.38 ± 0.02^b^
	P5	51.6 ± 1.07^c^	6.1 ± 0.17^c^	0.0 ± 0.00^a^	18.9 ± 1.08^a^	4.7 ± 0.30^b^	0.44 ± 0.06^b^
Heat +	WT	39.3 ± 0.90^d^	3.5 ± 0.24^e^	4.2 ± 0.22^d^	3.9 ± 0.53^d^	1.1 ± 0.11^e^	0.16 ± 0.02^e^
Drought	P1	41.0 ± 1.04^d^	4.2 ± 0.21^f^	2.6 ± 0.29^b^	6.3 ± 0.94^e^	1.2 ± 0.08^e^	0.16 ± 0.03^e^
	P4	39.3 ± 0.87^d^	4.3 ± 0.12^f^	2.0 ± 0.17^b^	6.6 ± 0.45^e^	1.0 ± 0.09^e^	0.16 ± 0.03^e^
	P5	41.7 ± 0.82^d^	4.7 ± 0.14^f^	2.3 ± 0.17^b^	5.9 ± 0.39^e^	1.0 ± 0.09^e^	0.12 ± 0.02^e^

*WT* wild-type; P1, P4 and P5, over-expression plants. Plants are those shown in Fig. 4 and measurements were performed five days after treatment

*Within each parameter, means ± SE ^a,b,c,d,e,f^ Same letter after values indicate that they are not significantly different from each other at p < 0.05

**Fig. 5:**
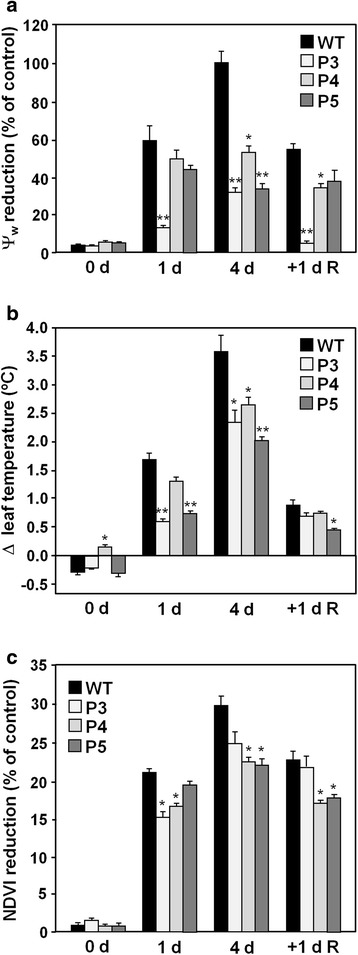
Physiological parameters of maize OsMYB55 transgenic plants subjected to drought stress in greenhouse conditions. Eight-week-old wild type (WT) and OsMYB55 lines (P3, P4, P5) grown in greenhouse were subjected to water withholding for five days and then re-watered as described in methods. **a** Reduction in water potential of stressed plants compared to well-watered controls. **b** Difference of leaf temperature in stressed and control plants. **c** Reduction of the normalized difference vegetation index (NDVI) values in stressed plants compared to controls. All measurements were performed in the upper, fully expanded leaves. Data shown are for 0, 1 and 4 days of no irrigation and 1 day after recovery. Bars represent mean values ± SE (*n* = 15). *P*-values (Student’s *t*-test) are indicated: **P < 0.005 and *P < 0.05

To further characterize the phenotype of the transgenic plants in response to drought in more advanced vegetative stages, an experiment was designed where the control of water availability was privileged over that of ambient temperature (i.e. greenhouse conditions). Eight-week-old plants were subjected to water withholding for five days and then allowed to fully recover, which was attained by all genotypes after 3–4 days. To assess the plants’ physiological condition, water potential (to determine water status), leaf temperature (indicative of leaf transpiration) and normalized difference vegetation index (NDVI; as an indicator of plant greenness or photosynthetic activity) were measured. Reduction of water potential occurred gradually in all genotypes but was slower in the OsMYB55 lines which lost between 30 and 50 % of water potential compared to that of well-watered plants after 4 days without irrigation, whereas WT plants lost twice as much water potential as their respective controls. This difference in water status remained significant in all three transgenic lines one day after recovery (Fig. 5a). The superior water status of the transgenic lines could be an indication of a higher transpiration rate compared to WT. This was further evaluated by measuring the depression of temperature in fully expanded leaves relative to well-watered plants. The OsMYB55 lines exhibited better evaporative cooling of the leaves than WT from the start of the water stress regime and presented up to 1.5 °C less increment in temperature compared to WT. This difference was less evident after recovery (Fig. 5b).

For assessing senescence and green area affected by stress, foliar reflectance was measured as another non-destructive phenotyping parameter. Based on the light absorbed in the visible versus the near infrared bands of the spectrum, several vegetation indices were calculated including simple ratio index, normalized difference vegetation index (NDVI), green NDVI, soil adjusted vegetation index, and triangular vegetation index. Because NDVI is the most common vegetation index used and is considered a sensitive indicator of green biomass, green leaf area and vigor [31], it was selected to illustrate the results (Fig. 5c). Reduction of the NDVI occurred right from the start of the experiment and was lower in the transgenic lines compared to WT. Those lines with significant less affected NDVI also exhibited better index during the early recovery phase (Fig. 5c). All four calculated indices produced similar trends and differences between the genotypes and throughout the treatment (not shown). Overall, the physiological parameters indicated that the water stress treatment affected WT plants more than OsMYB55 lines, suggesting increased tolerance to water deficit of the latter group.

## Discussion

Plant transcription factors are key regulators of almost all aspects of plant growth and development as well as for responses to stress conditions. HSFs are the major group of transcription regulators involved in HS response [8] however independent studies have consistently identified other groups of transcription factors that respond to HS in cereals, including the MYB-type family [25, 26, 32]. In this work, we show that over-expression of *OsMYB55* in maize improves plant tolerance to high temperature and water stress during vegetative growth and whole-genome transcript profiling identified transcripts and physiological processes that may contribute to their performance under stress.

### Heat stress and drought tolerant phenotype of OsMYB55 transgenic maize

Plant growth and yield depend on the efficiency of photosynthesis and metabolism to produce biomass. Under HS or drought, the integrity of the plant’s physiology is compromised by the onset of oxidative stress and alteration of water relations, causing reduction of photosynthesis and impairment of redox homeostasis [7]. The reduced leaf damage and MDA accumulation in OsMYB55 lines (Figs. 1 and 4) suggest that these transgenic plants may have enhanced cell membrane stability and ROS scavenging ability under HS conditions. This is likely due to the activation of endogenous ROS-scavenging systems or other defense responses (see below). Compared to C3 plants, maize is generally more capable of sustaining growth under high temperatures due to a greater antioxidant capacity. It has been reported that the expression and activities of ascorbate peroxidase, catalase, glutathione reductase and superoxide dismutase, as well as contents of reduced ascorbate and glutathione, are higher in maize plants compared to those in wheat and rice, before and after being exposed to HS [33, 34]. However, as for rice [30], an increase of 12–13 °C in temperature decelerated growth of maize plants. It should be noted that even though leaf damage was more pronounced in WT under combined HS and drought (Table 1), such conditions were exceedingly harsh for all genotypes, making recovery impossible. During HS, plants open their stomata to reduce leaf temperature through transpiration. However, when HS is combined with water deficit, plants tend to close stomata and the leaf temperature remains high [21]. Under drought, we found that OsMYB55 plants were able to maintain cooler leaves with a better water potential status than WT plants (Fig. 5), which would be a desired trait when both heat and drought are present in field conditions. The better capacity of the OsMYB55 transgenic lines to preserve a functional photosynthetic apparatus under a single stress condition may also reflect an improved antioxidant defense system against abiotic stresses.

The effects of HS and drought treatments were consistent in each case among all transgenic lines tested. However more significant differences in growth parameters and leaf damage were apparent between the OsMYB55 lines and WT plants under HS than in the drought assay (Table 1). It is possible that some of the genes activated by OsMYB55 are associated more with cellular functions for overcoming HS than with water deficit; although significantly less water loss was observed in the transgenic lines under HS (Fig. 4e). Nonetheless, reduced MDA accumulation in transgenic lines occurred in all stress conditions and the difference with WT plants was more pronounced under combined stress (Fig. 4f). This observation together with the regulation and possible roles of DEG in the transgenic lines suggest that both treatments may be triggering common physiological responses.

In general, cereals are more sensitive to environmental changes at the reproductive stage; hence more studies are focused on the mechanisms of heat response in reproductive tissues. Interestingly, a transcriptome analysis of rice panicle at anther developmental stage after heat treatment revealed that the response involve similar processes to those occurring in vegetative tissues (e.g. cellular homeostasis, transport, stress response and transcriptional regulation; [32]). The study also shows that in addition to the regulation of ROS-related genes, maintenance of ROS levels in rice panicles is also relevant to maintain ROS balance [32]. It would be interesting to evaluate the effect of HS in the reproductive tissues of OsMYB55 maize lines. Unfortunately, maize yield parameters are frequently unable to be assessed under greenhouse conditions and we were not able to field test these lines due to regulatory restraints.

### Transcriptome analysis of OsMYB55 maize: interpreting possible functional elements

GO analysis determined similar enrichment of stress-related biological processes in both transgenic and WT maize in response to HS (Fig. 2). To understand the improved tolerance in these lines, the genes associated with stress responses were analyzed. The analysis revealed that several up-regulated genes under normal conditions in transgenic maize were also highly up-regulated in WT plants in response to HS, and other stress-related genes were also significantly up-regulated in the transgenic line in the heat treatment comparison (Additional file 9). One essential group comprised transcription factors. Most type A and type B HSFs respond to HS, however some also are induced by drought, cold or salt stress [35, 36]. Analysis of DEG in the OsMYB55 transgenic line only identified one type B2 HSF (*HSFTF19*, AC216247.3_FG001) to be up-regulated both in control conditions and by HS (Additional file 9; Fig. 3). The same gene was reported to be induced by heat, drought and salt stress [36]. HSFs can also be activated by other transcription factors. It has been shown that a heat-induced HSFA3 in *Arabidopsis* is regulated by the AP2-type transcription factors DREB2A and DREB2B [37]. Despite a typical association of DREB transcription factors with dehydration and cold responses, cross regulation of DREBs with other signals seem to unravel new links between heat responses with drought and osmotic stress [37]. Among the DEG in the OsMYB55 transgenic line (Additional files 9 and 12), a C2H2 zinc finger protein, (ZOS11-01) has been shown to be induced by drought and to regulate DREB1b [38]. The list of DEG also includes three members of the AP2/EREBP transcription factor family (Additional file 10). WRKY transcription factors have also been described to contribute to tolerance to heat, drought, cold and salt stress in rice, wheat, barley and soybean (reviewed in [39]). In the OsMYB55 line, two WRKY proteins were up-regulated, but orthologs of only one (ZmWRKY120) have been described to be induced by stress and to confer drought tolerance when overexpressed [40]. But perhaps the most represented family of transcription factors that distinguishes the transgenic maize line from WT was the MYB group. Five genes encoding MYB proteins were up-regulated in the transgenic line in control conditions and four of them when the heat treatment was compared (Additional file 9), suggesting important participation of these regulators in the response to HS. Besides OsMYB55, only three other examples (in *Arabidopsis*, tomato and *Quercus*) have identified MYB factors as mediators of responses to HS [28, 29, 41]. MYBs have also been included among transcription factors affecting responses to water deficit [42]. Interestingly, none of the MYBs up-regulated in OsMYB55 maize or putative orthologs have been implicated in drought responses (Additional file 12). It is reasonable to presume that OsMYB55-induced protective actions against oxidative stress are also contributing against the negative effects caused by water deficit.

Among other gene classes, only one gene related to HSP (GRMZM2G032547) was identified as differentially expressed in the OsMYB55 line compared to WT, indicating that expression of most HSPs in response to HS were similar in both genotypes. GRMZM2G032547 encodes an ATP-dependent Clp protease which is related to chaperone HSP104 with a weak similarity to AtHSP101, a ClpB chaperonin required for thermotolerance [43]. A thaumatin (GRMZM2G038490) was highly up-regulated in OsMYB55 maize. Thaumatins are usually implied in plant biotic stress interactions but have also been reported to be induced by abiotic stress including heat [44]. Over-expression of a cotton thaumatin gene, *GbTLP1*, with a potential role in secondary cell wall development, showed enhanced resistance against different stresses including drought and salinity [45]. Another up-regulated gene encodes a wall-associated receptor-like kinase (*WAK*, AC217293.3_FG007). WAKs can function as links between intracellular compartments and the extracellular environment and are thought to have a role in cell elongation, development and stress responses [46]. Down-regulation of *WAK1* in rice affects root growth and produces dwarf plants and male sterility [47] whereas in barley it also affects root phenotypes in plants grown under control and stress conditions [48]. Other group of up-regulated genes in OsMYB55 maize may be more implicated in responses to oxidative stress, such as two lipid transfer proteins (LTPs, AC194203.3_FG003 and AC155352.2_FG010). LTPs are known for their involvement in stresses as well as biological processes such as cutin biosynthesis [49] though evidence on their role in heat stress is limited. A wheat *LTP3* gene has been shown to enhance oxidative stress and thermotolerance of *Arabidopsis* plants [50]. Transcript levels of a gene encoding an adenosine 5'-phosphosulfate reductase (APR, GRMZM2G028010) were also up-regulated in OsMYB55 maize and furthermore sulfate assimilation and phosphate acquisition were among the pathways enriched in the transgenic line under HS (Additional file 8). APR is a key enzyme of sulfate assimilation and its mRNA levels increases upon oxidative stress and salt treatment in a gibberellin-dependent manner [51]. Sulfur-containing compounds are involved in plant stress defense however little is known about the mechanisms of stress-regulated sulfate metabolism. It is plausible that sulfate metabolism may play a role in heat stress tolerance of OsMYB55 transgenic maize. The transcriptome analysis did not detect specific activation of the genes involved in amino acid metabolism reported for rice over-expressing *OsMYB55* [30], which points to differences in gene regulatory networks relevant for heat stress response between these two species.

Pathways involving hormones, antioxidants and osmo-solutes also contribute to thermotolerance [8]. For example, treatments with ABA, SA and 1-aminocyclopropane 1-carboxylic acid (ACC, an ethylene precursor) can reduce heat-induced reduce ROS accumulation and lipid peroxidation [52] and cytokinin levels also can modify the enzymatic antioxidant defenses against heat stress and drought [53]. It was shown that modulation of cytokinin levels influences the control of leaf water potential and stomatal conductance and leads to an enhanced heat and drought tolerance in tobacco plants [13]. In addition, expression of a cytokinin glycosyltransferase has been shown to improve drought stress adaptation [54]. GO terms related to cytokinin signaling (Fig. 2) and conjugation (glycosylation; Additional file 8) were among those enriched in OsMYB55 transgenic maize. Whether OsMYB55 mediates gene expression of hormonal signals that respond to stress remains to be investigated. Lastly, one of the highly heat-induced genes in OsMYB55 maize encodes a patatin-like phospholipase (AC194158.3_FG005) which belongs to a group of phospholipases that have been described as mediators of hormone signaling associated to responses to abiotic stress [55].

An interesting finding in the transcriptome analysis was the enrichment of GO terms related to DNA replication, nucleosome assembly and microtubule-based processes in the transgenic line (Fig. 2). These are important processes for cell division and gene regulation. Chromatin remodeling involving histone dynamics in nucleosomes can trigger transcriptional regulation of thermal response in plants (reviewed in [56]). A significant study on this topic described that, under high temperature, eviction of the histone H2A.Z from nucleosomes facilitates transcription of target genes involved in the thermosensory activation of flowering [57]. It is still unclear however, whether expression of heat-induced genes required for thermo-tolerance (e.g. HSFs and HSPs) can be regulated in the same fashion. Nonetheless, it was established that the same *H2A.Z* gene determines heat stress effects on grain yield in *Brachypodium* [58]. Recently, a gene encoding a tRNAHis guanylyl transferase (*Ica1*) was identified to be required for plant growth at high temperatures in *Arabidopsis. ica1* mutants present enhanced sensitivity to DNA damage, a defect in G2/M transition and down regulation of cell cycle genes under high temperatures [59]. Perhaps not surprisingly, of 52 up-regulated genes in OsMYB55 maize and with possible roles in drought tolerance (Additional file 12), five of them encode histones previously identified as drought responsive and six encode minichromosome maintenance complex (MCM) subunits which are involved in assuring complete and accurate DNA replication during each cell cycle and also implicated in abiotic stress responses [60].

## Conclusions

The transcriptome analysis of maize over-expressing *OsMYB55* revealed up-regulation of genes encoding proteins involved in general defense responses and abiotic stress, including HS and drought, suggesting that those plants may be using signaling pathways and genetic and epigenetic regulations common to responses to various stress conditions. Deciphering the molecular mechanisms underlying these functional genes will aid to better understand stress tolerance and to select strategies for improving crop productivity facing climate change scenarios.

## Methods

### Plant growth and treatments

Seeds of wild-type elite maize (*Zea mays*) inbred (SRG200, Syngenta) and transgenic lines over-expressing *OsMYB55* were germinated in Turface (calcined clay grains; International Minerals and Chemical, ON, Canada) for 7 days. Heat stress, drought and combined stress treatments were carried out in growth chambers. For these experiments, ten-day-old seedlings of similar height (48 seedlings per genotype) were transplanted into 1 L pots filled with peat moss and perlite (3:1; SunGro Horticulture, BC, Canada) and grown at 29 °C / 23 °C (12 h light, ~500 μmol m^−2^.s^−1^) for three more weeks. When the plants were about 5 week old, they were divided in four groups: two groups, each with 12 plants per genotype, were placed at 40 °C/34 °C for five days (HS treatment) and the other two groups (also 12 plants each) were left at 29 °C / 23 °C as controls. At the same time, one group in each temperature condition was subjected to water withholding for the same five-day period (drought treatment). After measurements were done, the remaining plants that were not sampled or destroyed were re-watered to observe recovery. To evaluate plant responses to drought at advanced vegetative stages (before they enter reproductive stage), a separate experiment was performed in greenhouse conditions. Thirty plants per genotype were grown in 5 gal pails filled with Turface for 8 weeks before the start of the drought experiment. This consisted in withholding water to half of the plants for five days and then they were re-watered to allow them to fully recover. Control plants were kept under well-watered conditions (15 plants per genotype). Plants were watered daily with a fertilized solution containing: 400 mg/L 15-15-30 High K soluble fertilizer, 400 mg/L 28-14-14 High N soluble fertilizer, 200 mg/L NH_4_NO_3_, 200 mg/L MgSO_4_.7H_2_O and 30 mg/L of chelated micronutrient mix (all nutrients supplied by Plant Products, ON, Canada). Plants were grown in a greenhouse under long-day regime with supplemented light (16 h light, ~500 μmol m^−2^.s^−1^) at 29 °C, and 8 h dark at 23 °C. In each experiment, the same initial volume of substrate and the same volume of water was applied to all pots.

### Construct preparation and plant transformation

A genetic construct to over-express *OsMYB55* was generated by cloning its full-length cDNA into a binary vector containing the *Ubi1* promoter [30]. Generation of transgenic lines was performed by Syngenta through *Agrobacterium tumefaciens*-mediated transformation and positive transformed plants were selected by the phosphomannose isomerase (PMI) test [61].

### Next generation sequencing

For RNAseq analysis, WT and transgenic (P4 line) plants were grown in chambers for 14 days and then half of them (the heat treatment group) were moved to 40 °C/34 °C for one week before collecting samples. Harvest of leaves (2nd and 3rd), stems (lower 4 cm) and whole roots was carried out at noon. Samples were collected separately from nine plants and pooled to obtain three pooled sample replicates. The plant materials were submerged in RNAlater (Ambion Inc., TX, USA) and stored at −80 °C until further analysis. All tissues were ground in liquid nitrogen and RNA was extracted using TRI-Reagent (Sigma-Aldrich, MO, USA) following the manufacturer’s instructions. Samples were treated with RQ1 RNase-free DNase (Promega, WI, USA) and total RNA was quantified using a Nanodrop 2000c spectrophotometer (Thermo Fisher Scientific, MA, USA). Samples were further processed at Syngenta for Next-Generation Sequencing (NGS) on an Illumina Hi-Seq 2000 platform. Single end reads of length 101 base pairs (bps) were collected. Reads were not trimmed and instead were filtered based on adapter contamination (i.e., if a read contained adapter sequences it was discarded). Reads were aligned to the Maize reference genome ZmB73_RefGen_v2 using GSNAP [62] with a set of known splice sites taken from Maize annotations 5b.60/ZmB73_5b_FGS. The alignment resulted in roughly >80 % of the filtered reads aligning to the reference genome. Next, reads aligned to annotated regions were counted. If a read overlapped with an annotated region of the genome it was counted. Lastly, the read counts were filtered using a reads-per-million strategy to reduce computation by excluding genes with no or very low signal. A minimum library size of approximately 19 million reads was used. Genes were filtered based on if a sample had less than a quarter of the reads per million, i.e., greater than 19/4 = 5 reads per million aligned to it. If at least 3 samples (three replicates) had more than 5 reads, then it remained in the dataset, otherwise it was removed. For data normalization, first, hierarchical clustering was used to analyze dissimilarities between samples. Next, full-quantile normalization (R package EDASeq version 1.4.0) that correct for GC content bias within and between samples was used to improve sensitivity without loss of specificity [63].

### Differential gene expression

Fold-change of DEG was calculated from normalized values using the R package edgeR version 3.0.4 [64]. This resulted in a set of P-values per gene indicating the statistical significance. To control the family wise error rate, P-values were then adjusted for multiple comparisons using the Benjamini-Hochberg method to produce a false discovery rate (FDR). Adjusted p-values or FDRs lower than 0.05 were considered statistically significant. Lists of DEG considered those with an FDR < 0.05 and a log2 fold change greater than 1 or less than −1.

### GO terms and pathway enrichment analyses

GO Term enrichment was performed using the R Bioconductor package GOseq (http://bioconductor.org/packages/release/bioc/html/goseq.html) which corrects for any bias induced by long, highly expressed transcripts [65]. Gene lengths were calculated using the maize ZmB73_5b_FGS gff3 file from maizegdb.org. In addition to gene lengths, GO Terms associated to Gramene gene IDs were pulled from Uniprot and Interpro databases. Once significant GO terms were identified, the P-values were corrected for family wise errors using the Benjamini-Hochberg method to calculate a FDR. FDRs less than 0.05 were considered significant. GOseq and the same methodology for GO Term enrichment was used to identify enriched pathways and pathways names were taken from MaizeCyc version 2.0.1. In this case, FDRs less than 0.1 were considered significant in order to capture more pathways.

### Real-time RT-PCR analysis

Total RNA was isolated from 100 mg of flash-frozen, pulverized leaves using the RNeasy Plant Mini Kit (Qiagen, CA, USA) and then treated with RQ1 RNase-free DNase (Promega). cDNA was synthesized using the qScript™ cDNA Synthesis kit (Quanta Biosciences, MD, USA). Quantitative real-time expression was performed using PerfeCTa SYBR Green SuperMix ROX (Quanta Biosciences) on an ABI7300 system (Applied Biosystems, CA, USA) using specific primers for selected genes (Additional file 13). Relative expression was calculated by the ^2–ΔΔ^Ct method [66] using the maize *18S RNA* gene (accession AF168884.1) as constitutive control. Analyses were performed with two technical and three biological replicates.

### Physiological and phenotypic parameters

For growth parameters (shoot height, root length, and plant biomass), twelve replicates were used for each measurement. Leaves were considered damaged when they presented dried or burnt tissue areas of any size. Other measurements included:

- *Chlorophyll content:* Total chlorophyll levels were measured in the middle section of a fully extended leaf of the same age using both the CCM-200 Chlorophyll Content Meter (Opti-Sciences, NH, USA) as well as the standard acetone extraction and spectrophotometeric quantification technique as described in [67].
- *Water status measurements:***R**elative water content (RWC) was determined as RWC = (fresh weight - dry weight)/(turgid weight - dry weight) × 100. Fresh weight was measured using a scale; turgid weight was determined by soaking leaf samples in distilled water, in darkness in a cold room (4 °C) for 24 h. Dry weight was determined by placing the same samples in an air oven at 70 °C until constant weight was reached. Leaf water potential was measured at midday in the first and second youngest fully expanded leaves using a potentiometer (model WP4, Decagon Devices, WA, USA) and leaf discs of 1-in. diameter that were removed with a leaf punch about 15 cm from the tip, avoiding the mid-vein.
- *Leaf temperature:* Leaf temperature was measured with a handheld infrared camera (model E60, FLIR, MA, USA). Measurements were taken around midday (11:00–14:00 h) in the same fully expanded leaves and consisted in the average temperature in a 5 cm square section of the mid-section of each leaf. The instrument was held at an angle of 30° to the horizontal plane and approximately 30 cm away from the target. Results are shown as temperature differences between stressed and well-watered plants.
- *Spectral reflectance:* An active canopy sensor, Crop Circle ACS-470 (Holland Scientific, NE, USA) with three interference filters covering green (550 nm), red (670 nm) and near infrared (NIR; 760 nm) wavebands regions was used to collect reflectance from the same leaves used for measuring other parameters. Output reflectance data of the sensor was set at a rate of 5 readings per second and approximately 30 readings were obtained to compute the average per individual leaf sample. The sensor was positioned perpendicular and at a distance of approximately 50 cm over the middle of the leaf. Reflectance data were recorded in a GeoSCOUT GLS-400 data logger (Holland Scientific) connected to the sensor and were used to compute the normalized difference vegetation index (NDVI) according to the formula: NDVI = (R_NIR_-R_R_)/(R_NIR_ + R_R_), where R_NIR_ and R_R_ represents the fraction of emitted NIR and red radiation returned from the sensed leaf area, respectively [68]. The three-channel reflectance data were also used to calculate other vegetation indices such as simple ratio index, green NDVI, soil adjusted vegetation index and triangular vegetation index [69].
- *Malondialdehyde (MDA) content*: MDA was determined by means of the thiobarbituric acid (TBA) reaction following the method described elsewhere [70] using 0.2 g of leaf tissue.

### Statistical analysis

Analysis of variance was performed using SigmaStat (SPSS Inc., IL, USA). Significant differences between treatment means were separated using the Tukey’s Honestly Significant Difference (HSD) test at α = 0.05.

## Availability of supporting data

Datasets supporting the results of this article are included in Additional files 3, 4 and 5.

## Additional files

Additional file 1: Transgene expression in over-expression lines. (PDF 48 kb) Additional file 2: Expressed genes and alignment percentages in all samples. (XLSX 12 kb) Additional file 3: List of all DEG in leaves. (XLSX 5263 kb) Additional file 4: List of all DEG in stems. (XLSX 5394 kb) Additional file 5: List of all DEG in roots. (XLSX 5556 kb) Additional file 6: Summary of differentially expressed genes by tissue between the conditions (heat stress and control). (PDF 85 kb) Additional file 7: Summary highlighting the differences in gene expression between wild type and OsMYB55 transgenic plants. (PDF 85 kb) Additional file 8: Enriched pathways represented in DEG. (PDF 181 kb) Additional file 9: Comparison of DEG: MYB55 line vs. WT and heat responsive. (XLSX 15 kb) Additional file 10: Common DEG in MYB55 line leaf control condition. (XLSX 12 kb) Additional file 11: DEG in MYB55 line leaf control condition associated to drought response/tolerance. (XLSX 16 kb) Additional file 12: Recovery phenotype from stress treatments of maize plants over-expressing *OsMyb55.* (PDF 209 kb) Additional file 13: Oligonucleotide primers used in real-time quantitative PCR. (XLSX 10 kb)

## References

[1] United Nations, Department of Economic and Social Affairs, Population Division (2011). World Population Prospects: The 2010 Revision, Volume I: Comprehensive Tables.

[2] Tai APK, Martin MV, Heald CL (2014). Threat to future global food security from climate change and ozone air pollution. Nature Climate Change.

[3] Stocker TF, Qin D, Plattner GK, IPCC (2013). Summary for Policymakers. Climate Change: The Physical Science Basis.

[4] Long SP, Ort DR (2010). More than taking the heat: crops and global change. Curr Opin Plant Biol.

[5] Barnabás B, Jäger K, Fehér A (2008). The effect of drought and heat stress on reproductive processes in cereals. Plant Cell Environ.

[6] Hatfield JL, Boote KJ, Kimball BA, Ziska LH, Izaurralde RC, Ort D, Thomson AM, Wolfe DW (2011). Climate impacts on agriculture: implications for crop production. Agron. J.

[7] Bita CA, Gerats T (2013). Plant tolerance to high temperature in a changing environment: scientific fundamentals and production of heat stress-tolerant crops. Front Plant Sci.

[8] Kotak S, Larkindale J, Lee U, Koskull-Doring P, Vierling E, Scharf KD (2007). Complexity of the heat stress response in plants. Curr Opin Plant Biol.

[9] Burke JJ, Chen J (2015). Enhancement of reproductive heat tolerance in plants. PLoS One.

[10] Chauhan H, Khurana N, Nijhavan A, Khurana JP, Khurana P (2012). The wheat chloroplastic small heat shock protein (sHSP26) is involved in seed maturation and germination and imparts tolerance to heat stress. Plant Cell Environ.

[11] Katiyar-Agarwal S, Agarwal M, Grover A (2003). Heat-tolerant basmati rice engineered by over-expression of *hsp101*. Plant Mol Biol.

[12] Wang D, Luthe DS (2003). Heat sensitivity in a bentgrass variant. Failure to accumulate a chloroplast heat shock protein isoform implicated in heat tolerance. Plant Physiol.

[13] Macková H, Hronková M, Dobrá J, Turečková V, Novák O, Lubovská Z, Motyka V, Haisel D, Hájek T, Prášil IT, Gaudinová A, Štorchová H, Ge E, Werner T, Schmülling T, Vanková R (2013). Enhanced drought and heat stress tolerance of tobacco plants with ectopically enhanced cytokinin oxidase/dehydrogenase gene expression. J Exp Bot.

[14] Clarke SM, Cristescu SM, Miersch O, Harren FJ, Wasternack C, Mur LA (2009). Jasmonates act with salicylic acid to confer basal thermotolerance in *Arabidopsis thaliana*. New Phytol.

[15] Khan MI, Iqbal N, Masood A, Per TS, Khan NA (2013). Salicylic acid alleviates adverse effects of heat stress on photosynthesis through changes in proline production and ethylene formation. Plant Signal Behav.

[16] Ogweno J, Song X, Shi K, Hu W, Mao W, Zhou YH, Yu JQ, Nogués S (2008). Brassinosteroids alleviate heat-induced inhibition of photosynthesis by increasing carboxylation efficiency and enhancing antioxidant systems in *Lycopersicon esculentum*. J Plant Growth Regul.

[17] Chan Z, Shi H (2015). Improved abiotic stress tolerance of bermudagrass by exogenous small molecules. Plant Signal Behav.

[18] Shi WM, Muramoto Y, Ueda A, Takabe T (2001). Cloning of peroxisomal ascorbate peroxidase gene from barley and enhanced thermotolerance by overexpressing in *Arabidopsis thaliana*. Gene.

[19] Tang L, Kwon SY, Kim SH, Kim JS, Choi JS, Cho KY, Sung CK, Kwak SS, Lee HS (2006). Enhanced tolerance of transgenic potato plants expressing both superoxide dismutase and ascorbate peroxidase in chloroplasts against oxidative stress and high temperature. Plant Cell Rep.

[20] Yang X, Liang Z, Lu C (2005). Genetic engineering of the biosynthesis of glycinebetaine enhances photosynthesis against high temperature stress in transgenic tobacco plants. Plant Physiol.

[21] Rizhsky L, Hongjian L, Mittler R (2002). The combined effect of drought stress and heat shock on gene expression in tobacco. Plant Physiol.

[22] Augustine SM, Narayan JA, Syamaladevi DP, Appunu C, Chakravarthi M, Ravichandran V, Subramonian N (2015). *Erianthus arundinaceus* HSP70 (*EaHSP70*) overexpression increases drought and salinity tolerance in sugarcane (*Saccharum* spp. hybrid). Plant Sci.

[23] Chauhan H, Khurana N, Agarwal P, Khurana JP, Khurana P (2013). A seed preferential heat shock transcription factor from wheat provides abiotic stress tolerance and yield enhancement in transgenic Arabidopsis under heat stress environment. PLoS One.

[24] Reddy PS, Kavi Kishor PB, Seiler C, Kuhlmann M, Eschen-Lippold L, Lee J, Reddy MK, Sreenivasulu N (2014). Unraveling regulation of the small heat shock proteins by the heat shock factor HvHsfB2c in barley: its implications in drought stress response and seed development. PLoS One.

[25] Qin D, Wu H, Peng H, Yao Y, Ni Z, Li Z, Zhou C, Sun Q (2008). Heat stress-responsive transcriptome analysis in heat susceptible and tolerant wheat (*Triticum aestivum* L.) by using Wheat Genome Array. BMC Genomics.

[26] Zhang X, Rerksiri W, Liu A, Zhou X, Xiong H, Xiang J, Chen X, Xiong X (2013). Transcriptome profile reveals heat response mechanism at molecular and metabolic levels in rice flag leaf. Gene.

[27] Ambawat S, Sharma P, Yadav NR, Yadav RC (2013). MYB transcription factor genes as regulators for plant responses: an overview. Physiol Mol Biol Plants.

[28] Feng C, Andreasson E, Maslak A, Mock HP, Mattsson O, Mundy J (2004). Arabidopsis MYB68 in development and responses to environmental cues. Plant Sci.

[29] Meng X, Wang JR, Wang GD, Liang XQ, Li XD, Meng QW (2015). An R2R3-MYB gene *LeAN2* positively regulated the thermo-tolerance in transgenic tomato. J Plant Physiol.

[30] El-Kereamy A, Bi YM, Ranathunge K, Beatty PH, Good AG, Rothstein SJ (2012). The rice R2R3-MYB transcription factor OsMYB55 is involved in the tolerance to high temperature and modulates amino acid metabolism. PLoS One.

[31] Araus JL, Serret MD, Edmeades GO (2012). Phenotyping maize for adaptation to drought. Front Physiol.

[32] Zhang X, Li J, Liu A, Zou J, Zhou X, Xiang J, Rerksiri W, Peng Y, Xiong X, Chen X (2012). Expression profile in rice panicle: insights into heat response mechanism at reproductive stage. PLoS One.

[33] Kumar S, Gupta D, Nayyar H (2012). Comparative response of maize and rice genotypes to heat stress: status of oxidative stress and antioxidants. Acta Physiol Plant.

[34] Stepien P, Klobus G (2005). Antioxidant defense in the leaves of C3 and C4 plants under salinity stress. Physiol Plant.

[35] Xin H, Qin F, Phan Tran LS (2012). Transcription factors involved in environmental stress responses in plants. Environmental Adaptations and Stress Tolerance of Plants in the Era of Climate Change.

[36] Yang Z, Wang Y, Gao Y, Zhou Y, Zhang E, Hu Y, Yuan Y, Liang G, Xu C (2014). Adaptive evolution and divergent expression of heat stress transcription factors in grasses. BMC Evol Biol.

[37] Larkindale J, Vierling E (2008). Core genome responses involved in acclimation to high temperature. Plant Physiol.

[38] Figueiredo DD, Barros PM, Cordeiro AM, Serra TS, Lourenço T, Chander S, Oliveira MM, Saibo NJ (2012). Seven zinc-finger transcription factors are novel regulators of the stress responsive gene *OsDREB1B*. J Exp Bot.

[39] Tripathi P, Rabara RC, Rushton PJ (2014). A systems biology perspective on the role of WRKY transcription factors in drought responses in plants. Planta.

[40] Luo X, Bai X, Sun X, Zhu D, Liu B, Ji W, Cai H, Cao L, Wu J, Hu M, Liu X, Tang L, Zhu Y (2013). Expression of wild soybean WRKY20 in Arabidopsis enhances drought tolerance and regulates ABA signalling. J Exp Bot.

[41] Almeida T, Pinto G, Correia B, Santos C, Gonçalves S (2013). *QsMYB1* expression is modulated in response to heat and drought stresses and during plant recovery in Quercus suber. Plant Physiol Biochem.

[42] Golldack D, Li C, Mohan H, Probst N (2014). Tolerance to drought and salt stress in plants: Unraveling the signaling networks. Front Plant Sci.

[43] Hong SW, Vierling E (2001). Hsp101 is necessary for heat tolerance but dispensable for development and germination in the absence of stress. Plant J.

[44] Zhang Y, Mian MA, Chekhovskiy K, So S, Kupfer D, Lai H, Roe BA (2005). Differential gene expression in *Festuca* under heat stress conditions. J Exp Bot.

[45] Munis MF, Tu L, Deng F, Tan J, Xu L, Xu S, Long L, Zhang X (2010). A thaumatin-like protein gene involved in cotton fiber secondary cell wall development enhances resistance against *Verticillium dahliae* and other stresses in transgenic tobacco. Biochem Biophys Res Commun.

[46] Wagner TA, Kohorn BD (2001). Wall-associated kinases are expressed throughout plant development and are required for cell expansion. Plant Cell.

[47] Kanneganti V, Gupta AK (2011). RNAi mediated silencing of a wall associated kinase, OsiWAK1 in *Oryza sativa* results in impaired root development and sterility due to anther indehiscence: Wall Associated Kinases from *Oryza sativa*. Physiol Mol Biol Plants.

[48] Kaur R, Singh K, Singh J (2013). A root-specific wall-associated kinase gene, *HvWAK1*, regulates root growth and is highly divergent in barley and other cereals. Funct Integr Genomics.

[49] Carvalho AO, Gomes VM (2007). Role of plant lipid transfer proteins in plant cell physiology-A concise review. Peptides.

[50] Wang F, Zang XS, Kabir MR, Liu KL, Liu ZS, Ni ZF, Yao YY, Hu ZR, Sun QX, Peng HR (2014). A wheat lipid transfer protein 3 could enhance the basal thermotolerance and oxidative stress resistance of Arabidopsis. Gene.

[51] Koprivova A, North KA, Kopriva S (2008). Complex signaling network in regulation of adenosine 5'-phosphosulfate reductase by salt stress in Arabidopsis roots. Plant Physiol.

[52] Larkindale J, Knight MR (2002). Protection against heat stress-induced oxidative damage in Arabidopsis involves calcium, abscisic acid, ethylene, and salicylic acid. Plant Physiol.

[53] Lubovská Z, Dobrá J, Storchová H, Wilhelmová N, Vanková R (2014). Cytokinin oxidase/dehydrogenase overexpression modifies antioxidant defense against heat drought and their combination in *Nicotiana tabacum* plants. J Plant Physiol.

[54] Li YJ, Wang B, Dong RR, Hou BK (2015). AtUGT76C2 an Arabidopsis cytokinin glycosyltransferase is involved in drought stress adaptation. Plant Sci.

[55] Scherer GF, Ryu SB, Wang X, Matos AR, Heitz T (2010). Patatin-related phospholipase A: nomenclature, subfamilies and functions in plants. Trends Plant Sci.

[56] Liu J, Feng L, Li J, He Z (2015). Genetic and epigenetic control of plant heat responses. Front Plant Sci.

[57] Kumar SV, Wigge PA (2010). H2A.Z-containing nucleosomes mediate the thermosensory response in Arabidopsis. Cell.

[58] Boden SA, Kavanova M, Finnegan EJ, Wigge PA (2013). Thermal stress effects on grain yield in *Brachypodium distachyon* occur via H2A.Z-nucleosomes. Genome Biol.

[59] Zhu W, Ausin I, Seleznev A, Méndez-Vigo B, Picó FX, Sureshkumar S, Sundaramoorthi V, Bulach D, Powell D, Seemann T, Alonso-Blanco C, Balasubramanian S (2015). Natural variation identifies *ICARUS1*, a universal gene required for cell proliferation and growth at high temperatures in *Arabidopsis thaliana*. PLoS Genet.

[60] Tuteja N, Tran NQ, Dang HQ, Tuteja R (2001). Plant MCM proteins: role in DNA replication and beyond. Plant Mol Biol.

[61] Negrotto D, Jolley M, Beer S, Wenck A, Hansen G (2000). The use of phosphomannose-isomerase as a selectable marker to recover transgenic maize plants (*Zea mays* L.) via *Agrobacterium* transformation. Plant Cell Rep.

[62] Wu TD, Nacu S (2010). Fast and SNP-tolerant detection of complex variants and splicing in short reads. Bioinformatics.

[63] Bullard J, Purdom E, Hansen K, Dudoit S (2010). Evaluation of statistical methods for normalization and differential expression in mRNA-Seq experiments. BMC Bioinformatics.

[64] Robinson MD, McCarthy DJ, Smyth GK (2010). edgeR: a Bioconductor package for differential expression analysis of digital gene expression data. Bioinformatics.

[65] Young M, Wakefield M, Smyth G, Oshlack A (2010). Gene ontology analysis for RNA-seq: accounting for selection bias. Genome Biol.

[66] Livak KJ, Schmittgen TD (2001). Analysis of relative gene expression data using real-time quantitative PCR and the 2-ΔΔCT method. Methods.

[67] Lichtenthaler HK, Wellbum AR (1983). Determinations of total carotenoids and chlorophylls a and b of leaf extracts in different solvents. Biochem Soc Trans.

[68] Tucker CJ (1979). Red and photographic infrared linear combinations for monitoring vegetation. Remote Sens Environ.

[69] Haboudane D, Miller JR, Tremblay N, Zarco-Tejada PJ, Dextraze L (2002). Integrated narrow-band vegetation indices for prediction of crop chlorophyll content for application to precision agriculture. Remote Sens Environ.

[70] Hodges DM, DeLong JM, Forney CF, Prange RK (1999). Improving the thiobarbituric acid-reactive-substances assay for estimating lipid peroxidation in plant tissues containing anthocyanin and other interfering compounds. Planta.

