# Supplementary material for: Expression of OsMYB55 in maize activates stress-responsive genes and enhances heat and drought tolerance
Source: BMC Genomics. 2016 Apr 29;17:312. doi: 10.1186/s12864-016-2659-5 (PMC4850646; doi:10.1186/s12864-016-2659-5)
Supplement: Additional file 1: — Transgene expression in over-expression lines. (PDF 48 kb) [file 12864_2016_2659_MOESM1_ESM.pdf]

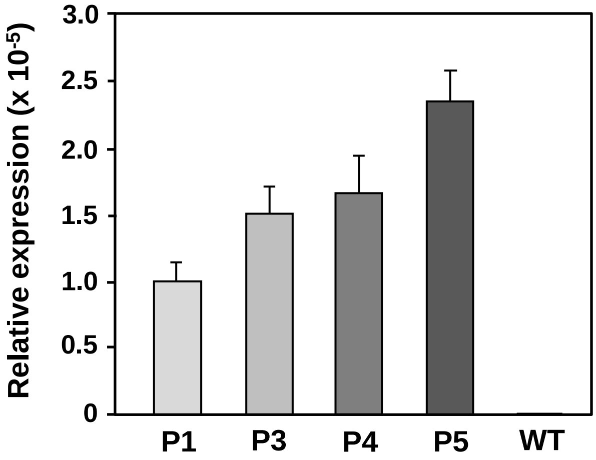

Additional file 1. Transgene expression in over-expression lines. Relative expression of OsMYB55 in over-expression lines. Bars represent means ± SE (three biological replicates and two technical replicates).
