# Supplementary material for: Expression of OsMYB55 in maize activates stress-responsive genes and enhances heat and drought tolerance
Source: BMC Genomics. 2016 Apr 29;17:312. doi: 10.1186/s12864-016-2659-5 (PMC4850646; doi:10.1186/s12864-016-2659-5)
Supplement: Additional file 6: — Summary of differentially expressed genes by tissue between the conditions (heat stress and control). (PDF 85 kb) [file 12864_2016_2659_MOESM6_ESM.pdf]

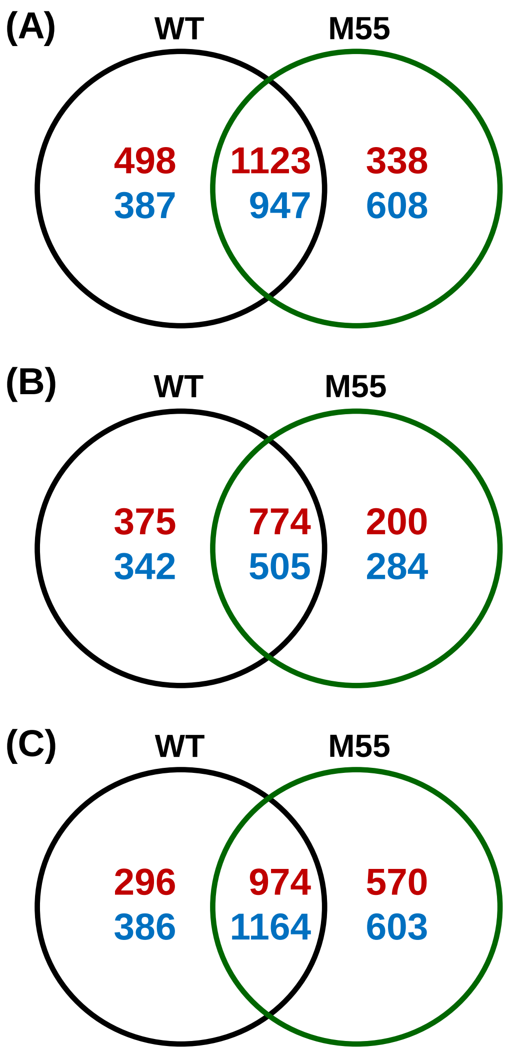

Additional file 6. Summary of differentially expressed genes by tissue between the conditions. Venn diagrams illustrate the number of genes which expression was altered by heat stress on either wild type (WT) or OsMYB55 transgenic plants (M55). The diagrams show sample comparisons between the conditions for (**a**) leaf, (**b**) stem and (**c**) root. Red and blue numbers indicate up- and down-regulated genes, respectively.
