# Supplementary material for: Expression of OsMYB55 in maize activates stress-responsive genes and enhances heat and drought tolerance
Source: BMC Genomics. 2016 Apr 29;17:312. doi: 10.1186/s12864-016-2659-5 (PMC4850646; doi:10.1186/s12864-016-2659-5)
Supplement: Additional file 7: — Summary highlighting the differences in gene expression between wild type and OsMYB55 transgenic plants. (PDF 85 kb) [file 12864_2016_2659_MOESM7_ESM.pdf]

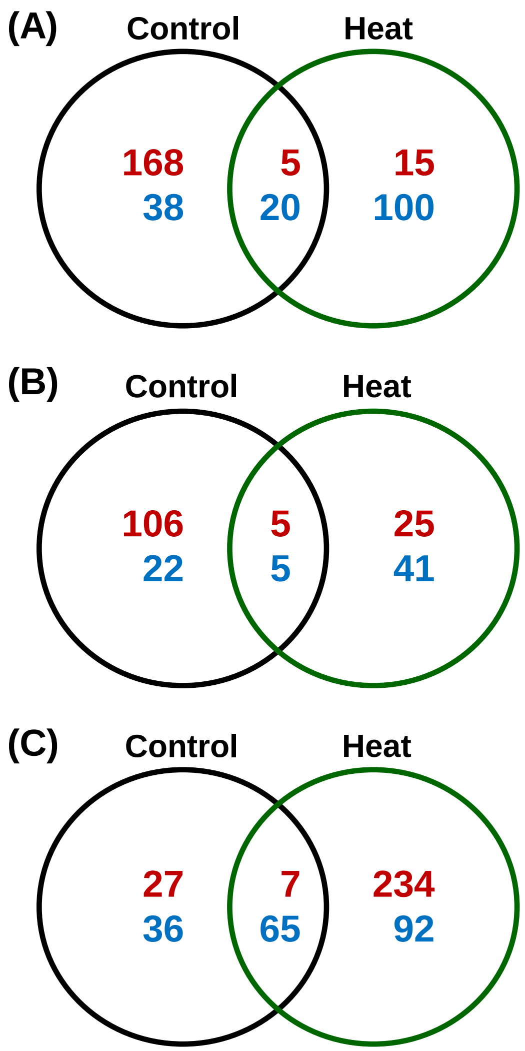

Additional file 7. Summary highlighting the differences in gene expression between wild type and OsMYB55 transgenic plants. The Venn diagrams illustrate the number of genes that show differences in expression between the plants under normal (Control) and heat stress conditions. The diagrams show sample comparisons between the genotypes for (**a**) leaf, (**b**) stem and (**c**) root. Red and blue numbers indicate up- and down-regulated genes, respectively.
