# Supplementary material for: Expression of OsMYB55 in maize activates stress-responsive genes and enhances heat and drought tolerance
Source: BMC Genomics. 2016 Apr 29;17:312. doi: 10.1186/s12864-016-2659-5 (PMC4850646; doi:10.1186/s12864-016-2659-5)
Supplement: Additional file 8: — Enriched pathways represented in DEG. (PDF 181 kb) [file 12864_2016_2659_MOESM8_ESM.pdf]

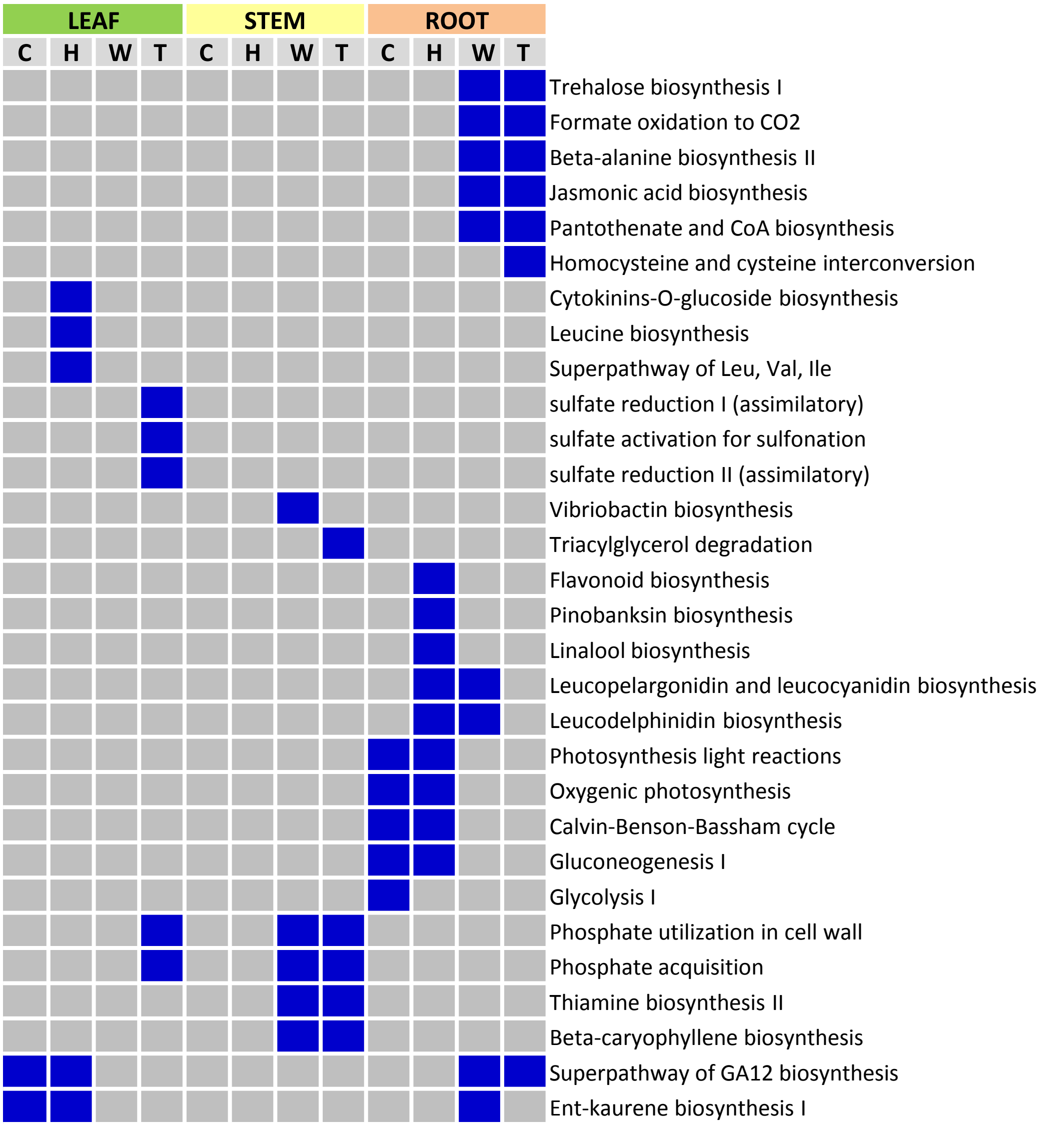

Additional file 8. Enriched pathways represented in DEG. Enriched pathways across tissue and sample categories (C, control; H, heat stress; W, wild type; T, transgenic line). Blue indicates statistical significance (FDR < 0.1).
