# Supplementary material for: Expression of OsMYB55 in maize activates stress-responsive genes and enhances heat and drought tolerance
Source: BMC Genomics. 2016 Apr 29;17:312. doi: 10.1186/s12864-016-2659-5 (PMC4850646; doi:10.1186/s12864-016-2659-5)
Supplement: Additional file 12: — Recovery phenotype from stress treatments of maize plants over-expressing OsMyb55. (PDF 209 kb) [file 12864_2016_2659_MOESM12_ESM.pdf]

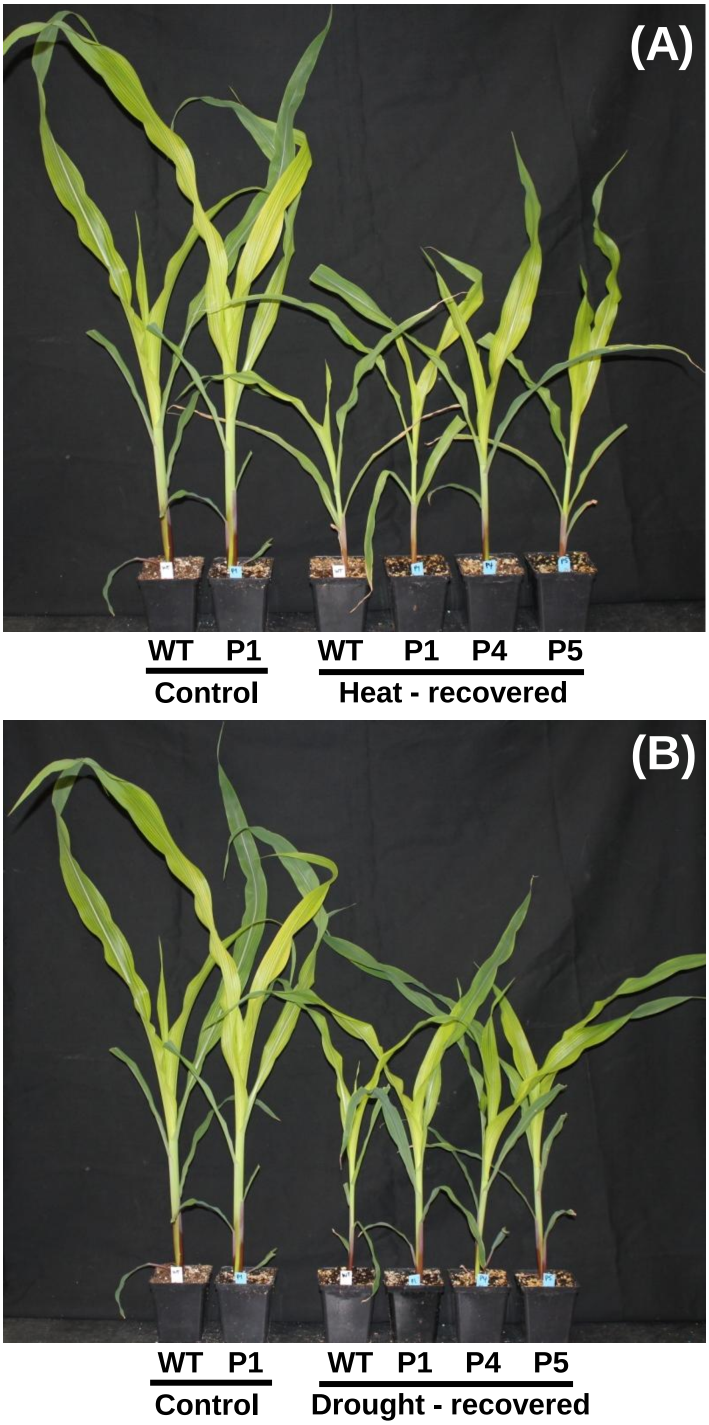

Additional file 12. Recovery phenotype from stress treatments of OsMYB55 transgenic maize*.* (**a**) Wild type (WT) and transgenic plants (P1, P4 and P5) grown under control conditions (see methods) or subjected to high temperature (42 ºC / 35 ºC, day/night cycle) for five days. Picture was taken seven days after shifting heat-treated plants back to normal temperatures (29 ºC / 23 ºC, day/night cycle). Control plants were watered and maintained at normal temperatures during the whole experiment. (**b**) WT and transgenic plants subjected to water withholding for five days and then recovered by watering for seven days.
